# Knowledge needs in patients with Liver Disease: a qualitative study

**DOI:** 10.1186/s12912-023-01580-7

**Published:** 2023-10-30

**Authors:** Birgitte Gade Jacobsen, Mette Munk Lauridsen, Lea Ladegaard Grønkjaer

**Affiliations:** Department of Gastroenterology, University Hospital of South Denmark, Finsensgade 35, Esbjerg, 6700 Denmark

**Keywords:** Knowledge needs, Liver Disease, Patient experience, Qualitative study, Unmet needs

## Abstract

**Background:**

Knowledge is essential for patients’ disease management strategies and a critical component of healthcare. The importance of increasing patients level of knowledge has become more widely acknowledge in liver disease management in recent years, but further studies are needed to address patients experiences of unmet knowledge needs to develop appropriate patient education strategies. Therefore, the aim of this study was to explore knowledge needs in patients’ with liver disease of different etiology and severity.

**Methods:**

A qualitative study was designed and an inductive method was chosen. Thirty-three patients with liver disease of different etiology and severity were interviewed using a semi-structured interview guide. Content analysis was used as an inspiration to describe and compare patients’ needs for knowledge across disease etiology and severity. The reporting followed consolidated criteria for reporting qualitative research.

**Results:**

The analysis generated three categories and nine subcategories. In general, the patients described lack of knowledge related to their liver disease, which made it difficult for them to manage their disease. Patients wished to be more involved in care and treatment of the liver disease. However, patients’ had difficulties to assess and understand the importance of the information they received from healthcare professionals. Due to lack of knowledge, patients’ had a misconception of the liver disease. Patients’ had variation in knowledge needs depending on liver disease etiology and severity.

**Conclusion:**

Within liver disease management, knowledge of patients’ experiences is vital to meet patients’ knowledge needs and to develop appropriate patient education strategies. Therefore, it is important to ascertain a patient-centered approach to accommodate patients’ individual knowledge needs, involve patients in care and treatment, and insure understanding to strengthen their self-management and give the patients the necessary skills to manage their disease and everyday life.

**Registration number:**

Open Science Framework registration DOI 10.17605/OSF.IO/W28RC.

## Background

Liver disease is commonly caused by alcohol abuse, metabolic dysfunction-associated steatohepatitis, viral hepatitis, and immune system abnormalities and may progress to cirrhosis [1]. Living with a liver disease can have a major impact on the patients´ life, and result in serious mental health problems such as anxiety, depression, stress, social isolation, change of body image and physical complications and symptoms such as ascites, fatigue, hepatic encephalopathy, impaired mobility, irregular sleep pattern, jaundice, muscle cramps, and varices. This affects patients’ everyday life and places considerable demands on their knowledge and management of the disease [2].

The management of the liver disease partly depends on the underlying etiology, but the goal is to prevent worsening of the disease, treat complications, and to avoid or delay clinical decompensation and transplantation [1]. Therefore, patients with liver disease needs to be empowered and motivated to self-manage [3]. However, studies have shown that patients with liver disease generally have low knowledge about the disease, and many patients consider liver disease management challenging due to required lifestyle modifications, medical adherence, and nutritional prescription [4–7]. This is further compounded by the nature of liver disease, where management becomes more complex as the disease progresses [8].

Knowledge is essential for patients’ disease management strategies and a critical component of healthcare. Studies have shown that the more the patients know about their disease, the more likely they are to engage in self-management and take responsibility for their own health, which results in an increased likelihood of concordance with care and treatment, reduced healthcare use, more positive health outcomes, and improved quality of life [3, 9].

The importance of increasing patient education and increasing patients level of knowledge has become more widely acknowledge in liver disease management in recent years, but further studies are needed to address patients experiences of unmet knowledge needs to develop appropriate patient education strategies [9, 10]. Therefore, the aim of this study was to explore knowledge needs in patients’ with liver disease of different etiology and severity.

## Methods

This study has an exploratory design with a qualitative inductive approach. Semi-structured interviews was performed and content analysis was used as an inspiration to explore and compare the knowledge needs of patients with liver disease across disease etiology and severity [11]. Such an approach is suitable when studying experiences, and when the research area is limited. In addition, the epistemology of this study is based on the premise that patients’ subjective and varying experiences are valid and in key respects may increase healthcare professionals’ understanding of patients’ needs for knowledge. The study was registered in the Open Science Framework with registration DOI 10.17605/OSF.IO/W28RC, and reporting followed consolidated criteria for reporting qualitative research [12].

### Participants

The participating patients were recruited from the outpatient clinic at the Department of Gastroenterology, University Hospital of South Denmark, Esbjerg. Inclusion criteria included patients regardless of age and gender with an established diagnosis of liver fibrosis or cirrhosis regardless of etiology and severity. Exclusion criteria were difficulty speaking or understanding the Danish language, mental illness, and overt hepatic encephalopathy. Twenty-six patients were invited to participate. Twenty-three accepted and three declined because of fatigue (n = 2) or lack of time (n = 1). Patients’ clinical and demographic characteristics are summarized in Table 1.

**Table 1:** Clinical and demographic characteristics of the participating patients (n = 23)

**Clinical features**	
Age, years, median (interquatile range)	60 (23–81)
Men / women	69% / 31%
Caucasian	100%
**Etiology of liver disease**	
Alcohol-associated	55%
Autoimmune hepatitis	10%
Alpha 1 antitrypsin deficiency	5%
Metabolic dysfunction-associated steatohepatitis	25%
Viral hepatitis	5%
Time since diagnosis, years, median (interquartile range)	2 (0.5-6)
**Severity of liver disease**	
Child-Pugh score, median (interquartile range)	6 (5–10)
Model of End-stage Liver Disease, median (interquartile range)	6 (7–17)
Decompensated cirrhosis	55%
Current alcohol use	20%
**Family status**	
Single / divorced / widower	32%
Married / cohabiting	68%
**Occupation**	
Working	25%
Sick leave	10%
Unemployed	15%
Disability pensioner	10%
Retired	40%

### Data collection

Patients were provided with written information, invited to participate in the study, and be interviewed in connection with an appointment at the outpatient clinic. To avoid patients feeling pressured to participate, none of the authors was involved in the care and treatment of the participating patients. The interviews lasted for an average of 40 min (range 25–60 min) and were performed in a secluded room by the first or last author. Clinical and demographic data were collected at the beginning of each interview. Patients were interviewed until no additional or new information regarding their needs for knowledge could be identified, which is consistent with an understanding of data saturation [13]. The interviews were conducted in the period March 2020 to June 2022. All interviews were taped and transcribed verbatim by the first and last author. A total of 182 pages emerged after transcription.

### Interview guide

To obtain in-depth knowledge of the patients’ needs for knowledge, a semi-structured interview guide was developed. The topics and questions for the interviews were determined in advance, but the structure was left open, allowing the patients to provide detailed narratives about their experiences [14]. The interview guide consisted of both broad and specific questions that centered on having a liver disease, concerns, consequences, and the need for knowledge as experienced by the patients. Probing questions were posed to encourage the patients to elaborate on and explain their experiences and needs to access deeper aspects of the phenomenon. At the end of each interview, patients were given the opportunity to provide additional comments. The interview guide was pilot tested twice, which resulted in minor linguistic adjustments. The pilot interviews were included in the analysis.

### Analysis

Qualitative analysis was performed in a systematic process with inspiration from content analysis [11]. First, an overview of the transcriptions was obtained through repeated readings of the texts. Subsequently, the experiences and understandings of each patient were read more systematically, line by line, for the purpose of identifying patterns (i.e. meaning units). Meaning units answering the study aim were then chosen, condensed, and provided with a code, closely reflecting the text content. The surrounding text was kept to preserve the context. The codes were sorted into exhaustive and mutually exclusive categories and subcategories, each comparing and describing specific types of experiences regarding needs of knowledge across disease etiology and severity. NVivo software (NVivo qualitative data analysis software; QRS International Pty Ltd. Version 12, 2018) was used for initial sorting of data. Thereafter, an alteration in between software and manual analysis was performed to facilitate the process and create an overview of the data. The meaning units and categories were subsequently re-read to ensure the validity and accuracy of the entire original data set [11, 15]. Table 2 illustrates the steps of the analysis. The analysis was performed by the first and the last author and the result discussed in the author group. Interviews and analysis were performed in Danish. Verbatim quotations were translated into English by the authors.

**Table 2:** Examples of the analysis steps

Category	Problems related to information about the liver disease		
Subcategory	Information from healthcare professionals		
	Meaning unit	Condensed meaning unit	Code
	I just do not understand what he is saying	Difficulties in understanding information from physician	Trouble understanding

### Ethical considerations

In accordance with the Helsinki Declaration, the patients were assured of confidentiality and the voluntary nature of participation throughout the process. The study process was explained to the participating patients. They were also informed about their right to withdraw from the study at any time, and that the interview transcriptions were presented at group level. Oral and written informed consent was obtained from all participating patients before the interview. The patients did not receive any financial support for participating in the study. Data storage was notified to the Region of Southern Denmark and was handled in accordance with the EU´s General Data Protection Regulation (GDPR), 2016/679.

## Results

The analysis process resulted in 83 codes sorted into three categories and nine subcategories concerning patients knowledge needs related to the liver disease, to maintain everyday life, and understand information regarding the liver disease (Table 3). Quotes selected from the original transcript are provided as examples to illustrate the identified categories and subcategories.

**Table 3:** Categories and subcategories exploring knowledge needs in patients’ with liver disease

Need for knowledge related to the liver disease	Need for knowledge related to maintain everyday life	Problems related to information about the liver disease
• Understanding the diagnosis • Perceptions of the liver disease • Being able to react on symptoms and complications of the liver disease • Different knowledge needs	• Living a normal life • Support from others	• Information from healthcare professionals • Search for information • Problems accommodating information

### Need for knowledge related to the liver disease

***Understanding the diagnosis*** Patients described that the diagnosis often was a surprise and a by-product of routine health checks or treatment for other comorbidities.

“It was a surprise. I did not see it coming… And what does it even mean?” (Informant 23).

Often patients suffered from other comorbidities, which they were more concerned about. When there were no signs or symptoms, the patients did not perceive the liver disease diagnosis as serious. Patients highlighted a need for basic knowledge about where the liver is located in the body, the normal function of the liver, and a detailed description of the liver disease diagnosis, and the course of the disease explained in a way they could understand.

***Being able to react on symptoms and complications of the liver disease*** Patients with fibrosis or compensated cirrhosis described the liver disease as an invisible disease, largely because they did not feel any pain or had no symptoms, which they could associate with the disease.

“I do not feel anything. It is like the disease is not there. Except when I am in hospital, of course.” (Informant 6).

They believed that knowledge on signs and symptoms of the liver disease would make them more alert on the disease and enable them to improve disease management and engage with lifestyle modifications.

Even patients with decompensated cirrhosis had difficulties in understanding how a disease in the liver could affect the brain function or cause fluid in the stomach, which made it difficult for them to acknowledge the association between the disease and the complications and act on changes. They highlighted additional knowledge to seek prevention of complications or treatment in good time.

***Perceptions of the liver disease*** Due to lack of knowledge about the different causes of liver disease, patients’ had misperceptions of the liver disease. Patients with a nonalcohol- associated liver disease could not understand why they were diagnosed with a liver disease; they thought it only happened to people who consumed a lot of alcohol. Even patients that had been diagnosed with a liver disease due to autoimmunity several years ago and received adequate care and treatment still could not understand the diagnosis and expressed a lack of knowledge of the diagnosis.

“I just do not get it. My doctor says it is my body that is attacking my liver. But still … I have hardly touched alcohol.” (Informant 13).

Patients with an alcohol-associated liver disease also had a misconception of the liver disease and needed further knowledge about the relationship between the amount of alcohol and the risk of developing a liver disease. Patients described having consumed alcohol, but nevertheless did not feel alcohol dependent and had a difficult time perceiving themselves as someone with an alcohol-associated liver disease.

“Yes, I have been drinking alcohol. However, I still do not understand. I have never consumed excessive amounts of alcohol. How can I get a liver disease?” (Informant 1).

***Different knowledge needs*** There was a greater need for knowledge in connection with the diagnosis, when the liver disease progressed, and when the patients had lived with the disease for a time and realized that they had received insufficient information at diagnosis.

However, depending on the liver disease etiology and severity patients’ had different knowledge needs. Thus, patients with fibrosis or compensated cirrhosis called for information on lifestyle modifications and how to improve their liver condition or prevent their liver disease from getting worse, while patients with decompensated cirrhosis wanted information on the course of the disease, prevention, and treatment of complications. Patients said that such information would be valuable to them and help them manage their disease and maintain a normal everyday life.

“I think information about something I can manage myself is extremely important, for example, which symptoms I need to be aware of.” (Informant 16).

### Need of knowledge related to maintain everyday life

***Living a normal life*** All patients with liver disease, regardless of severity, wished to continue to live a normal everyday life. The patients required knowledge about how to behave in everyday life in order not to cause deterioration in their condition. Patients had received advice from the healthcare professionals for example to eat healthier, exercise, lose weight, or stop drinking alcohol but still needed knowledge on strategies to carry out these lifestyle modifications.

Difficulties in maintaining everyday living occurred as the severity of the liver disease increased. Patients described having fatigue, itching, loss of appetite and energy, and sleep problems. As the patients lacked basic knowledge about the liver disease, some of the patients were in doubt as to whether it was due to the liver disease or other comorbidities and how to address these problems, and information from healthcare professionals was limited.

“I do not think we ever talked about it. The physician only talks about blood tests and my medicine.” (Informant 5).

Living a normal life also required being social with other people. This was associated with a feeling of stigmatization. The liver disease caused patients to feel ashamed due to lack of knowledge in the general population and the perception that liver disease always is the result of an unhealthy lifestyle and therefore self-inflicted. It made patients refrain from telling others about their disease.

“I do not tell anyone about my liver disease. It is not their business, and I do not want people to talk ill of me.” (Informant 14).

Due to limitations, especially in severe stages of the liver disease, patients had to adjust to a new life with fewer physical and social activities, which resulted in social isolation. The limitations also affected patients’ mental health and made them depressed, sad, and worried about the disease progression.

“Most of the time, I am just at home. I feel quite alone… I think a lot. What is going to happen? Will I feel even worse?…I get sad thinking about it. (Informant 20)

Some patients expressed that talking with healthcare professionals or sharing experiences with other patients could be helpful.

***Support from others*** Emotional support from relatives was of great importance for the patients, and enabled the patients to better accept and live with the liver disease.

“I do not know what I should do without my wife. She means everything to me.” (Informant 1).

If the relatives also had knowledge about the liver disease and were engaged in the care and treatment, it was easier for the patients to achieve concordance with treatment and lifestyle modifications. Some patients experienced that their relatives had a greater need for knowledge regarding the disease than they themselves did, and were more concerned as the disease progressed.

“My wife wants to know everything… She worries about me all the time.” (Informant 18).

Patients with severe stages of liver disease expressed becoming more dependent on relatives and healthcare professionals due to physical limitations.

“I do not do anything at all… I cannot do anything without help from others.” (Informant 18).

They expressed need for knowledge on support services to support them and their relatives and perhaps provide them with tools to live their lives as normally as possible.

### Problems related to information about the liver disease

***Information from healthcare professionals*** All patients had talked to a physician in connection with diagnosis, but the consultation time was short, and it was difficult for the patients to assess the importance of the information they received.

“Well, sometimes people are in a hurry. And I do not want to trouble him (the physician) with questions … But sometimes I just do not understand what he is saying.” (Informant 10).

The patients did not feel that anyone had followed up on that talk or that the information given was directed at them personally leaving them with several knowledge needs regarding for example lifestyle modifications to prevent worsening and progression, signs or symptoms to be aware of, or the treatment of the liver disease and the associated complications. In addition, it left them with a feeling of not being involved in their own care and treatment of their disease.

“Well, he did not really ask me what I thought about it.” (Informant 7).

Some patients had received written patient information about the disease, but they had not necessarily read it.

“Honestly, I have not read what they gave me … I do not know why … Maybe I just do not think I will understand it anyway.” (Informant 1).

***Search for information*** Although the patients experienced knowledge needs, only very few of the patients sought information themselves.

“I do not know if I can trust the information on liver diseases available on the Internet.” (Informant 21).

In addition, they would not call the department if they had questions regarding the liver disease and were uncertain about symptoms and treatment, as they did not want to be a burden to the healthcare system.

***Problems accommodating information*** A few patients stated that they had problems accommodating the information they received from the healthcare professionals. They did not want information about their liver disease because they were afraid to find out how sick they really were and what would happen when the liver disease progressed. Patients believed that such knowledge might make them feel even sicker.

“I do not want to know the prognosis.… I do not want to think about it.” (Informant 14).

## Discussion

This study used a qualitative approach to explore the needs for knowledge in patients’ with liver disease of different etiology and severity. These results clearly indicate that patients lack liver disease knowledge, which influenced their ability to manage the disease. We found, that patients needed basic knowledge about the normal function of the liver and the liver disease. Patients knowledge needs were individual and varied according to liver disease etiology and severity. Thus, patients with fibrosis or compensated cirrhosis called for information on lifestyle modifications and how to improve their liver condition or prevent their liver disease from getting worse, while patients with decompensated cirrhosis wanted information on the course of the disease, prevention, and treatment of complications. Due to lack of knowledge, patients’ had a misconception of the liver disease.

The results of our study are in accordance with other studies, which describe that patients with liver disease generally have low knowledge of the disease and need information in regards to the liver disease such as disease cause and prognosis, medical aspects, and treatment as well as self-management [5–7, 16, 17]. However, our study also reveals that there is a need for knowledge in areas besides these, such as the great variation in knowledge needs depending on liver disease etiology and severity and being involved in care and treatment of the liver disease. In addition, our study also disclose that it was difficult for the patients to assess and understand the importance of the information they received from healthcare professionals.

Limited knowledge has been identified as a significant barrier to cope with chronic disease [18]. Our and other studies have shown that patients have a great interest in learning more about their liver disease, indicating a need for more educational programs in liver disease management [7, 16]. Two small studies have shown that liver disease education classes or videos improves the knowledge of patients and their relatives [19, 20]. However, providing the patient with information on the disease may no necessary lead to changes in behavior that will in turn lead to lifestyle modifications and improved self-management. Self-management is the individual´s ability to manage symptoms, treatment, physical and psychosocial consequences, and lifestyle changes inherent in having a chronic disease” [21]. A large number of self-management interventions have been developed for patients with other chronic diseases such as arthritis, asthma, and diabetes resulting in increased concordance with care and treatment, reduced healthcare use, more positive health outcomes, and improved quality of life [22]. Two small studies in patients with cirrhosis has shown promising results with increased cognitive level, health behaviors, and quality of life. However, further studies are needed to investigate the effect of self-care interventions in patients with liver disease [22, 23].

Patients knowledge needs varied depending on liver disease etiology and severity. It remains uncertain which knowledge interventions are the most beneficial in meeting patients’ knowledge needs in all stages of the liver disease. Current strategies include written patient information and patient schools with lectures [7]. In addition, the patients did not feel that the information given was directed at them or followed up, which resulted in a feeling of not being involved in the care and treatment of the liver disease. It emphasizes, along with the patients different knowledge needs, a patient-centeredness with a focus on providing a liver disease care plan with individualized information [24]. This requires a good patient-healthcare professional relationship and adequate time. Thus, it has been suggested that nurses could be more involved and play a role in the education and support of patients and their families through nurse-led consultations of longer durations than consultations by physicians, thus improving patients’ knowledge and reducing the burden on the healthcare system [25]. In addition, approaches include implementation of communication tools to facilitate patient participation such as advance care planning or shared decision-making to help patients understand and make decisions about their liver care, providing healthcare professionals with advanced communication skills, and using digital or social media in the healthcare system could improve disease knowledge and self-management [26, 27]. Although many different factors are likely to have affected the patients’ wish to gain more knowledge, some patients had no interest in receiving further information in regard to their liver disease. This can be considered a coping, self-protection mechanism, and it has been proposed that in such situations it is important that the healthcare professionals adjust the information to the patient’s coping style [28].

We found that patients had difficulties in assessing and understanding the importance of the information they received from healthcare professionals. An emerging area in the field of improving patient knowledge is health literacy, that is, the capacity to find, understand and act on health information [29]. Health literacy is an often overlooked factor in chronic disease management and needs integration in best practices to improve self-management. The results of our study may indicate that these patients had limited health literacy. This is supported by other studies demonstrating poor health literacy in patients with cirrhosis [30, 31]. The “teach-back” communication methods has been shown to improve knowledge in patients with chronic hepatitis B. The methods encourages healthcare professionals to ask patients to explain, in their own words, the information that has been covered. If the patient is unable to recall or has difficulty understanding the information, the healthcare professional can identify specific misunderstandings and re-explain the concept [32].

Patients described that support from relatives or healthcare professionals played a role in helping them manage their liver disease and maintain their everyday life. It is well known that support is linked to better disease management and health outcomes in patients with chronic diseases [33]. Thus, care for liver patients should aim to offer individualized support driven by a formal assessment of patients’ needs rather than by assumptions regarding prognosis. Moreover, relatives are an important, but often overlooked source of support to facilitate optimal disease management and should be included by healthcare professionals.

Patients had misconceptions due to lack of knowledge of liver disease. It affected patients’ life negatively and prevented them from telling others about their disease. This is consistent with previous studies of patients with liver disease [34, 35]. Even the patients themselves associated liver disease with an unhealthy lifestyle and excess drinking. This may be a substantial barrier to accepting the diagnosis and seeking support [36]. Hence, healthcare professionals working with patients with liver disease must be aware of these perceptions and their impact on patients’ interaction with the healthcare system. In addition, programs such as the Lancet-EASL Commission on liver disease together with governmental and non-governmental organizations, patient representatives, and experts in ethics and human rights are necessary to overcome inequities, stigmatization, and unmet needs in patients with liver disease [37].

### Strength and limitations

A strength in this study is the relatively large number of patients; as the sample size in qualitative studies is usually smaller. To increase the reliability of the study, pilot interviews were conducted to ensure that the interview guide was relevant and properly formulated. Validity was established by presenting relevant quotations from the transcribed text to further illustrate the categories. Discussions between the authors throughout the analysis process also contributed to the validity of the results. The detailed description of the study aim, participating patients, data collection, and analysis enables the reader to assess the transferability of the results [11].

However, there are several limitations. First, the interviews were performed at the hospital and the environment might not have be convenient for the patients. Second, the patients were interviewed right after they had agreed to participate. However, more preparation time may have led the patients to recall and reflect more on their experiences. Third, the patient were included from one department, which may not provide variations of data; although in qualitative methods, variation is not related to demographic characteristics but to variations of experiences [39]. Besides these limitations, this study may be a useful contribution to the sparse literature on knowledge needs in patients with liver disease, and be precedent for further studies within this area.

## Conclusion

In conclusion, this qualitative study has identified a previously unknown understanding of the impact of knowledge needs in patients with liver disease. Within liver disease management, knowledge of patients’ experiences is vital to meet patients’ knowledge needs and to develop appropriate patient education strategies. Therefore, it is important to ascertain a patient-centered approach to accommodate patients’ individual knowledge needs, involve patients in care and treatment, and insure understanding to strengthen their self-management and give the patients the necessary skills to manage their disease and everyday life.

## Data Availability

The dataset generated during and/or analyzed during the current study are available from the corresponding author on reasonable request.

## References

[1] Tsochatzis EA, Bosch J, Burroughs AK (2014). Liver Cirrhosis. Lancet.

[2] Grønkjær LL, Lauridsen MM. Quality of life an unmet need in patients with chronic liver disease: a mixed-methods systematic review. Submitted to JHEP Reports 2021. 10.1016/j.jhepr.2021.100370 PMC8585663 34805816

[3] Clayton M, Fabrellas N, Luo (2021). From NAFLD to MAFLD: nurse and allied health perspective. Liver Int.

[4] Wainwright SP (1997). Transcending chronic Liver Disease: a qualitative study. J Clin Nurs.

[5] Volk ML, Fisher N, Fontana RJ (2013). Patient knowledge about Disease self-management in Cirrhosis. Am J Gastroenterol.

[6] Burnham B, Wallington S, Jillson IR (2014). Knowledge, attitudes, and beliefs of patients with chronic Liver Disease. Am J Health Behav.

[7] Saleh ZM, Bloom PP, Grzyb K (2020). How do patients with Cirrhosis ad their caregivers learn about and manage their health? A review and qualitative study. Hepatol Commun.

[8] Hayward KL, Martin JH, Cottrell NW (2017). Patient-oriented education and medication management intervention for people with decompensated Cirrhosis: study protocol for a randomized controlled trial. Trials.

[9] Hayward KL, Valery PC, Patel PJ (2020). Effectiveness of patients-oriented education and medication management intervention in people with decompensated Cirrhosis. Intern Med J.

[10] Ghamdi [SSA, Shah H (2018). An educational needs assessment for patients with Liver Disease. J Can Assoc Gastroenterol.

[11] Krippendorf K (2018). Content analysis: an introduction to its methodology.

[12] Tong A, Sainsbury P, Craig J (2007). Consolidated criteria for reporting qualitative research (COREQ): a 32-item checklist for interviews and focus groups. Int J Qual Health Care.

[13] Patton MQ (2002). Qualitative research and evaluation methods.

[14] Kvale S, Brinkmann S (2018). Doing interviews.

[15] Graneheim UH, Lundman B (2004). Qualitative content analysis in nursing research: concepts, procedures and measures to achieve trustworthiness. Nurse Educ Today.

[16] Ghamdi SSA, Shah H (2018). An educational needs assessment for patients with Liver Disease. J Can Assoc Gastroenterol.

[17] Hjorth M, Svanberg A, Sjöberg D (2020). Liver Cirrhosis turns life into an unpredictable roller coaster: a qualitative interview study. J Clin Nurs.

[18] Low JTS, Rohde G, Pittordou K (2018). Supportive and palliative care in people with Cirrhosis: international systematic review of the perspective of patients, family members and health professionals. J Hepatol.

[19] Kadokawa Y, Katayama K, Takahashi K (2017). The effectiveness of a Liver Disease education class for providing information to patients and their families. J Clin Med Res.

[20] Goldsworthy MA, Fateen W, Thygesen H (2017). Patient understanding of liver Cirrhosis and improvement using multimedia education. Frontline Gastroenterol.

[21] Barlow J, Wright C, Sheasby J (2022). Self-management approaches for people with chronic conditions: a review. Patient Educ Couns.

[22] Zandi M, Adib-Hajbagheri M, Memarian R (2005). Effects of a self-care program on quality of life of cirrhotic patients referring to Tehran Hepatitis Center. Health Qual Life Outcomes.

[23] Zhang X, Xi W, Liu L (2019). Improvement in quality of life and activities of daily living in patients with liver Cirrhosis with the use of health education and patient health empowerment. Med Sci Monit.

[24] Chaudhary N, Lucero C, Villanueva G (2017). Assessment of abilities of gastroenterology fellows to provide information to patients with Liver Disease. Clin Gastroenterol Hepatol.

[25] Fabrellas N, Carol M, Palacio E (2020). Nursing care of patients with Cirrhosis: the liverhope nursing project. Hepatology.

[26] Grønkjær LL, Berg K, Søndergaard R (2020). Assessment of written patient information pertaining to Cirrhosis and its Complications: a pilot study. J Patient Exp.

[27] Austin CA, Mohottige D, Sudore RL (2015). Tools to promote shared decision making in serious Illness: a systematic review. JAMA Intern Med.

[28] Nordin K, Lidén A, Hansson M (2002). Coping style, psychological distress, risk perception, and satisfaction in subjects attending genetic counselling for hereditary cancer. J Med Genet.

[29] Nutbeam D (2008). The evolving concept of health literacy. Soc Sci Med.

[30] Grydgaard MF, Bager P (2018). Health literacy levels in outpatients with liver Cirrhosis. Scand J Gastroenterol.

[31] Kaps L, Hildebrand K, Nagel M (2021). Risk factors or poorer health literacy in patients with liver Cirrhosis. PLoS ONE.

[32] Tran S, Bennett G, Richmond J (2019). Teach-back is a simple communication tool that improves Disease knowledge in people with chronic hepatitis B- a pilot randomized controlled study. BMC Public Health.

[33] Huh J, Ackerman MS. Collaborative help in chronic disease management: supporting individualized problems. CSCW Conf Comput Support Coop Work 2012; 2021:853–862. 10.1145/2145204.2145331 PMC4211623 25360442

[34] Vaughn-Sandler V, Sherman C, Aronsohn A (2014). Consequences of perceived stigma among patients with Cirrhosis. Dig Dis Sci.

[35] Baker MS, McWilliam CL (2003). How patients manage life and health while waiting for a liver transplant. Prog Transpl.

[36] Fouad Y, Waked I, Bollipo S (2020). What’s in a name? Renaming NAFLD to MAFLD. Liver Int.

[37] Manns MP, Burra P, Sargent J (2018). The Lancet-EASL Commission on Liver Diseases in Europe: overcoming unmet needs, stigma, and inequities. Lancet.

